# Participatory Rapid Appraisal and Focus Groups to co-design technology-supported integrated care

**DOI:** 10.1371/journal.pone.0299411

**Published:** 2026-06-16

**Authors:** Bridget O’ Sullivan, Paul Davis, Regina Connolly, Shane O’ Hanlon, Marije Hamaker, Hans Wilders, Cindy Kenis, Una Kearns, Trudy Corrigan, Ciara White, Eleonore Lehn, Marianne Gros, Lucia Ferrara, Vittoria Ardito, Celia Fourrier, Pierre Soubeyron, Siri Rostoft, Vincent Thevenet, Anthony Staines

**Affiliations:** 1 Faculty of Science and Health, School of Nursing, Psychotherapy, and Community Health, Dublin City University Dublin, Ireland; 2 Dublin City University Business School, Dublin City University, Dublin, Ireland; 3 Dublin City University Business School, Dublin, Ireland; 4 University College Dublin - National University of Ireland, Dublin, Ireland; 5 Utrecht University, Utrecht, Netherlands; 6 KU Leuven University: Katholieke Universiteit Leuven, Leuven, Belgium; 7 myPatientSpace, Digital Healthcare developer and provider, Dublin; 8 School of Policy and Practice, Institute of Education, Dublin City University, Dublin, Ireland; 9 School of Nursing, Psychotherapy, and Community Health, Dublin City University, Dublin, Ireland; 10 University Bordeaux, Bordeaux, France; 11 lGi Sustainalbe Innovations, Paris, France; 12 Bocconi University, Milan, Italy; 13 Institute Bergonie, Bordeaux, France; 14 Oslo University Hospital Internal Medicine Research Unit: Oslo Universitetssykehus Enhet for Indremedisinsk Forskning, Oslo, Norway; 15 Centre eIntegrated Care (CeIC), Dublin City University, Dublin, Ireland; University of Oxford Nuffield Department of Clinical Medicine: University of Oxford Nuffield Department of Medicine, UNITED KINGDOM OF GREAT BRITAIN AND NORTHERN IRELAND

## Abstract

**Background:**

Co-design methods, which create innovation tailored to the end-user needs and setting, are increasingly used to improve research uptake and impact. Peer review and methodologists have emphasised the need for early, meaningful, and continuous involvement of stakeholders, and the transparent and detailed reporting of co-design methods. The implementation of both complex interventions (such as an integrated care pathway), and technology, into healthcare is difficult and has high failure rates. *Designing, implementing, and adapting interventions* to ensure they work in the local context requires in-depth understanding of multiple end-users’ needs, processes, and contexts. Achieving this requires early, meaningful, and continuous engagement between multiple end-users. This study aimed to contribute to co-design research by reporting the: 1) process used to develop and test Participatory Rapid Appraisal and Focus Groups methods to co-design integrated care. 2) details of how the method was applied in the GERONTE project. 3) core steps involved in this co-design method (to facilitate use and/ or adaptation of the method).

**Aim:**

The study aimed to develop, test, and evaluate a co-design method that: I. enabled early, continuous, and meaningful engagement of multiple end-users in different locations and across different design iterations. II. enabled timely feedback and checking between the end-users and design team. III. optimised end-users participation by being flexible in the time, duration, and method of data collection and feedback.

**Materials and methods:**

The Participatory Rapid Appraisal and Focus Group to co-design integrated care method was developed in four stages. The first stage involved defining the project’s co-design needs and reviewing the co-design literature to identify how to meet these. GERONTE aimed to co-design, evaluate, and prepare for EU-wide deployment, an integrated technology-supported care pathway for older adults with cancer and other morbidities. Focus Groups and Participatory Rapid Appraisal, in combination, were chosen as an empirically-based and practical approach. Focus Groups (FG) provided a participatory-based way to collect data in order to identify and agree multiple stakeholders’ needs and collective priorities. Participatory Rapid Appraisal (PRA) provided a timely way; to gain participant feedback on the data collected; and, to ensure accuracy in the data sent to the pathway and technology design teams. The second stage involved applying the method in the GERONTE Project (using meetings between the project team, technologists, and older adults to develop a co-design protocol for the project). The third stage involved the use, refinement, evaluation, and reporting of the co-design method. The fourth stage, evaluation of the method, is ongoing.

**Results and discussion:**

This study resulted in the development of a structured approach to using Participatory Rapid Appraisal and Focus Groups in combination to co-design technology-supported integrated care. This method is proposed as an evidenced-based, practical, user-friendly way to co-design *(or adapt an existing design)* an integrated technology-supported care pathway. This co-design method involves three cycles of design. Each cycle involves multidisciplinary FG to collect semi-structured data followed by rapid analysis and feedback of the FG data to the participants to design or refine the intervention.

## Introduction

### Need for integrated care

Healthcare policy, research and management have aimed to improve the quality and efficiency of services while responding to patient calls for more personalised and patient-centred care, and clinicians’ calls for organisational and technological support to achieve this [1,2,3,3,4,5]. Increased healthcare demands and costs, resulting from changing life and health expectancy [6], are intensifying the demand for sufficient, sustainable, accessible care [EU 7,8] that addresses chronic illness, multi-morbidity, and complex needs in a patient-centred cost-efficient way [9]. Person-centred care requires; integration and coordination across multiple disciplines, services, and sites; and, flexibility to respond to patients’ evolving care and support needs [10,11].

There is broad agreement that integrated care results in improved safety, quality, and value [12,13]   Considerable research and investment have not achieved the needed or sustained adoption, scale-up, and spread of integrated or patient-centred models of care due to the difficulty and complexity in designing and adapting interventions so they achieve the intended outcomes when implemented [14,15].

### Co-designing and implementing integrated care

Designing and implementing complex interventions, such as integrated pathways, or technology, into existing healthcare systems has a high failure rate. The local context (population needs, resources, policies, and processes) impact if and how the intervention works. Common and critical causes of failure include the: complexity in creating interventions than can meet multiple patients’ varied and complex needs; costs and limited resources; and conflict with existing organisational processes or practices [16,17]. Most complex interventions, even those evidenced as effective, require adaptation to local contexts to achieve the intended outcomes [15].

There are a number of key steps and processes to implementing integrated care, including; identifying, formally involving, and supporting the specialist medical, health, and support professionals that need to be involved in the care pathway; identifying, and making available, the health and patient information (needed by patients and health professions during the patient’s care journey); and appointing and supporting a dedicated role, ideally a nurse specialist, as a central point of contact for the patient, and to co-ordinate the care across services. The professionals, process, time, and support needed will differ in line with the population’s, the illnes’, and sites’ needs [9]. Providing integrated care at scale is resource- and time-intensive, often requiring technological innovations to make it feasible. Digital technologies can support structured communication, data collection and sharing (for improved decision-making), the provision of tailored supportive information (based on patient’s profile), and automated alert and follow-up systems to support coordination of care at scale  [18,19]. Co-design methods and Implementation Science (IS) are distinct and rapidly developing areas, with the shared aim of creating sustainable interventions that are tailored to the local needs and contexts, and that can result in real-world impact [20, Cherly et al, 2022, 21].

Co-design involves meaningful collaboration between researchers, health service users and providers, and other stakeholders, to identify, prioritise, co-design, test, implement and evaluate solutions. Co-design principles (inclusion, participation, outcome-focussed) have been widely adopted across health and social science disciplines, with extensive evidence of positive outcomes [22]. Commonly used co-design methods in healthcare include focus groups, surveys, expert groups, design workshops, user-testing and feedback, and public events [21]. While there is a significant amount of research reporting the use and outcome of co-design, and an increasing amount of research that describes in detail the data collection, analysis, synthesis methods, there is limited empirical evidence *specific to* the co-design of technology-supported integrated pathway which are increasingly part of healthcare reform [23,21].

Implementation Science has established evidence-based theories, models, frameworks (TMF), and strategies [21]. Increasingly these highlight the need to consider the impact of context on the intervention and its implementation [24,25]. Classic theories (such as theories on behavioural or change), process models (such as those outlining the process or phases of translation of knowledge or research into practice), determinant frameworks (outlining factors that influence implementation and adoption), and implementation theories, collectively provide a comprehensive understanding of IS. While *the use* of TMF, such as the classic “Theory of Diffusion” [26], the “Consolidated Framework for Implementation Research” [27], the process model “Knowledge to Action” [28], the implementation model “Organisational Readiness” [29], and the Re_AAIM Framework [30]  have grown, and resulted in improved implementation success rates. In order to facilitate replication and evaluation of methods in other contexts, research methodologies and peer-reviewers has advocated for improved and detailed reporting on how the IS or co-design method enables early, meaningful, continuous, and pre-implementation participant involvement and impact [31,32] and support practical and sustainable solutions to end-users’ needs [33].

Current co-design research outputs demonstrate; methodological strength in the identification, selection, inclusion, and representation of the diverse cohorts of relevant stakeholders [22,34,35,21,36,37]. They also demonstrate philosophical alignment between co-design principles and data collection, analysis, and synthesis methods. Co-design method commonly use a combination of survey, focus or working groups, and/ or product testing to co-design the product or interventions. As co-design methods are increasingly used and developed, researchers and end-users are calling for evidence of continuous (in addition to early) stakeholder involvement across design iterations, and an increased level of detail on the methods [34,22], as failure to maintain stakeholders’ engagement can result in late-stage design changes, delays, or failure at the pilot or implementation stages [22,38,39]. From an IS perspective, a co-design method that facilitates early, continuous, and cross-stakeholder engagement provides a rich and robust dataset to identify and document end-users’ needs and contexts, enabling evaluation against a meaningful baseline. It also enables identification of areas needing further exploration to support and inform implementation [40,15]. Co-designing an intervention, with the involvement of the necessary broad base of stakeholders working in busy healthcare systems, requires a robust, but pragmatic, iterative way to collect, analyse, and synthesise data to design and refine the intervention so that it meets the end-users needs and context [16,40]. Detailed reporting of methods supports peer-review and scientific development and refinement of methods in addition to support replication of studies and research efficiency [41,42,43–45].

### Co-designing an integrated technology-supported care pathway as part of the GERONTE project

As part of the GERONTE study, the project team identified a need for an empirically-based, practical, and replicable method to co-design (and later adapt) the GERONTE intervention (to support widespread uptake).

GERONTE **combined Focus Group (FG)** and **Participatory Rapid Appraisal (PRA)**, **in a structured way (three design cycles of data collection, analysis, feedback and design)** as its co-design method.

Each design cycle involved collection and analysis of semi-structured data from multidisciplinary stakeholders in Focus Groups. This was followed by ‘rapid analysis’ of the collected data to produce a summary of end-users’ needs and wants. The summary was checked by the FG participants for accuracy and comprehensiveness. Once approved, the summary was sent to the design team. This ensured the early and continuous involvement of the end-users in the intervention design and refinement.

#### Focus groups.

Focus Group (FG) is a qualitative research data collection method that facilitates in-depth exploration of the collective view on a pre-defined topic through open communication and the dynamic interaction between participants [46,47]. While the questions are determined by the research aim, the discussions are usually semi-structured. The FG size and duration is determined by the aim and stage, questions, and population needs.

FG are practical and advantageous in the design of an integrated technology-supported care pathway, as they facilitate the discussion between multiple stakeholders to achieve collective agreement on needs, priorities, and practical limitations. FG are also practical and flexible methods within busy health and research projects, as they are familiar to many disciplines, require unsophisticated resources (private comfortable room, seating, recording devices) that are available in most clinician and research facilities, and they can be conducted at flexible times to meet participants’ needs [48].

#### Participatory rapid appraisal.

Participatory Rapid Appraisal (PRA) draws from early Implementation Science methods, where it was used effectively in International Development Aid and agriculture, to provide success where many other costly interventions had failed at, or following, implementation [49]. A PRA approach *advocates collaboration with local people; in identifying priority problems; in collecting detailed contextual data* (using a variety of data collection methods such as in-depth interviews, observation, or ethnography); and, in identifying and developing *sustainable interventions* aligned to participants’ needs, priorities, personal and cultural preferences, and locally available and sustainable inputs [49,50]. PRA emphasises facilitation, empowerment, local knowledge and actionable, equitable and sustainable decisions and interventions, and it opposes a top-down, linear approach to decision making, and so it facilitates the development and implementation of interventions suited to, and sustainable, in different contexts.

In practical terms, PRA supports qualitative, quantitative, or mixed methods of data collection, analysis, synthesis, and reporting, but directs the methods to ensure *meaningful involvement* (participation) of stakeholders, and *rapid analysis* of their *needs, priorities, and resources,* with the aim of co-designing and refining a sustainable, practical solution [51].

### Reporting on the GERONTE co-design method

This manuscript aims to contribute by sharing for review, and/ or adaptation and use, the development, application, and steps in the GERONTE Participatory Rapid Appraisal and Focus Groups in combination to co-design technology-supported integrated care. Table 1 provides an overview of the type of information presented in this manuscript and how it is structured.

**Table 1:** Overview and structure of the information presented in this manuscript.

	What this manuscript presents	Relevant section(s) of the manuscript	Purpose
**1**	The process used to develop and test the co-design method	The Material and Methods section provide a summary of the steps used to develop, test, and refine the co-design method. S1 Appendix (in the Supplementary Materials) describes the complete process.	To facilitate peer review and feedback on the method’s theoretical background, development, and testing, and refinement.
**2**	Details of how method was applied in the GERONTE Project	The Material and Methods, in particular “Stage 2” provides a summary of how the co-design method was applied in GERONTE. S2 Appendix (in the Supplementary Materials), the “GERONTE Co-design Protocol/ Guide”, provides the full details of how the method was applied.	To facilitate peer review of the method’s application, strengths, limitations, and outputs. To provide a worked example.
**3**	The core steps in the method	The Results section provides an overview of the steps in the GERONTE PRA and FG to Co-design Itechnology-supported ntegrated Care..	To facilitate use and/ or adaptation in other projects.

## Materials and methods

### Developing, applying, and refining the Co-design method in GERONTE

The Material and Method section identifies the process used to develop, apply, and refine *Participatory Rapid Appraisal and Focus Groups* to Co-design Integrated Care in the GERONTE Project*. The GERONTE clinicians and management (oncologists and geriatricians, nurse specialist and the multidisciplinary team, patient representatives, and implementation team):

1. co-designed the GERONTE care pathway
2. collaborated with technologists to develop and adapt technology to support that care pathway.

The implementation team researched, recorded, and reported the GERONTE co-design process.

The *Participatory Rapid Appraisal and Focus Groups* to Co-design Integrated Care was developed in four stages, which is described briefly below and in more detail in S1 Appendix (Supplementary Material). These were:

1) Collaboration between the GERONTE stakeholders to identify the project’s co-design aims and needs (the timelines, stakeholders involved, and the resources needed and available) and a rapid literature review to identify a suitable co-design method
2) Stakeholder meetings to develop the method and ‘GERONTE co-design protocol (S2 Appendix)’
3) Apply, test, refine, and report the co-design method in the GERONTE project
4) Continued evaluation of the method.

*An EU-funded GERONTE project (GA945218) which aims to co-design, test, and prepare for EU-wide deployment an integrated technology-supported care pathway for older adults with cancer and other morbidities.

### First stage: Identify the co-design needs and a suitable method

**Fig 1:**
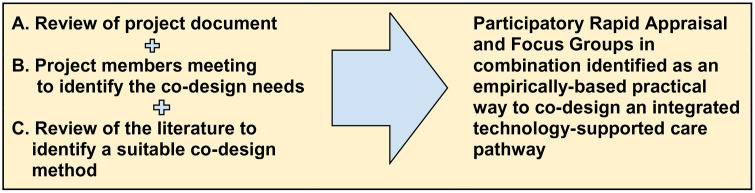
Overview of the process used to identify a suitable co-design method.

The first stage (Fig 1), involved collaborative meetings to identify the project’s co-design aims and needs. This was followed by a review of the literature to identify a suitable co-design method. S1 Appendix (Supplementary Materials) provides a detailed overview of the first stage.

#### Project documents and meetings were used to identify and agree the co-design needs.

The data sources used to identify the co-design aims, needs, and timelines were:

a) key GERONTE documents (EU-Grant Agreement and governing documents) that identified the aims, timelines, outputs, and the research and reporting requirements and
b) feedback from the project stakeholders.

The document and meetings data were analysed and synthesised thematically and discussed across meetings to agree collective and priority aims, needs, and timelines (that the co-design method needed to support).

The stakeholders agreed that GERONTE’s co-design aims and needs were to:

- ensure early, continuous, and meaningful involvement of multiple different categories of stakeholders from different locations and within a project’s time limits
- co-design an integrated technology-supported care pathway that would meet end-users’ needs and contexts.

As such, a co-design method was needed that:

- facilitated the inclusion and discussion between multiple stakeholders in different locations across a number of design iterations
- enabled rapid analysis and feedback between the researcher, end-users, and design team to enable refinement across design iterations
- was sufficiently flexible to adapt to stakeholders’ time schedules.

#### Literature review to identify a suitable method.

A review of the literature identified FG and PRA in combination met GERONTE’s co-design needs. FG provided an empirically-based practical way to collect semi-structured data from multiple stakeholders about their needs and wants. PRA provided an empirically-based practical way to rapidly gain participant feedback to refine the intervention.

The data sources and process involved a multidisciplinary research panel that conducted a search of CINAHL, MEDLINE, SCOPUS and Google Scholar databases between the search dates 2010 and 2022, limited to empirical research published in the English language, using the terms and string:

1) (‘Co-design’ OR ‘co-creation’ OR ‘co-production’ AND ‘methods’) AND
2) (‘Implementation Science’ AND ‘theor*’ OR ‘model*’ OR ‘framework*) AND
3) ‘healthcare’,

to identify, analyse, and summarise the literature on co-design and Implementation Science principles and practices.

**Brief description of GERONTE’s core aims: a) improve care, outcomes, and experiences ([as defined by pre-defined objective clinical and health measurement], b) be economically viable [using a cost-benefit analysis to identify the total benefits as compared to the total costs], c) meet end-users needs, and d) be sustainable, scalable, and adaptable [as determined by the end-user feedback and implementation evaluation]).

### Second stage: Developing the method and protocol

**Fig 2:**
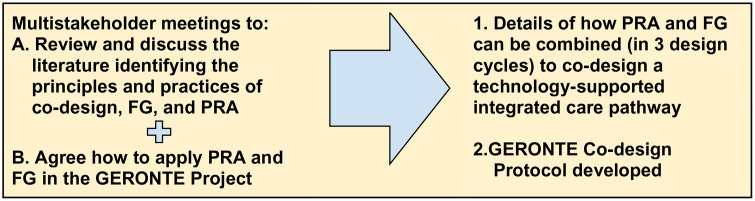
Overview of the process used to develop the PRA and FG co-design method and protocol.

The second stage (Fig 2), was a sequence of multi-stakeholders meeting (between clinicians, research methodologists, patient representative groups, and managers) to:

- consider the suitability, strengths, and weaknesses of the proposed, and other, methods
- develop the ‘co-design protocol’ to be used in the GERONTE’s project.

#### Co-design, FG, and PRA literature and multistakeholder meeting were used to develop the method.

Following agreement on use of PRA and FG as a co-design method, a protocol describing how to use PRA and FG to co-designthe GERONTE intervention (and/ or to and/ or adapt it to additional sites) was developed. The ‘GERONTE co-design protocol’ is presented in S2 Appendix (Supplementary Material), but in summary it:

1. documents *GERONTE’s* co-design aims
2. defines the participant recruitment and inclusion processes (for the FG), and
3. detail the practical tasks and processes required for the FG data collection and rapid analysis (PRA) and synthesis needed to co-design an integrated technology-supported care pathway.

The data sources and processes used to develop the protocol included;

1. literature providing guidance on co-design, PRA, and FG principles and practices
2. sequential multi-stakeholder meeting (each with specific aims, outcome measures, and timelines) to develop, review, and refine the protocol across meetings (to ensure that it was robust, practical, reproducible, and achieved the intended aims).

### Third stage: Applying and refining the method (in GERONTE)

**Fig 3:**
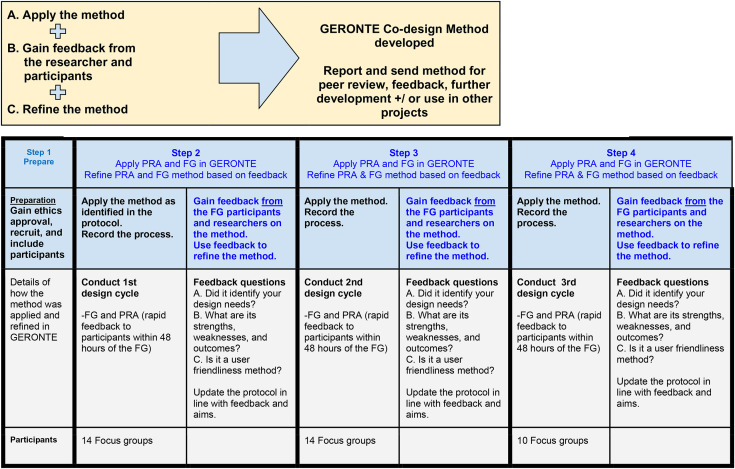
A. Overview of the process used to apply and refine the PRA and FG co-design method. B. Detailed view of the steps in applying and refining the PRA and FG co-design method.

The third stage involved applying and refining the method in GERONTE. It was an iterative process (Fig 3A and 3B), which is described briefly below, and presented in full detail in S1 Appendix.

**Fig 4:**
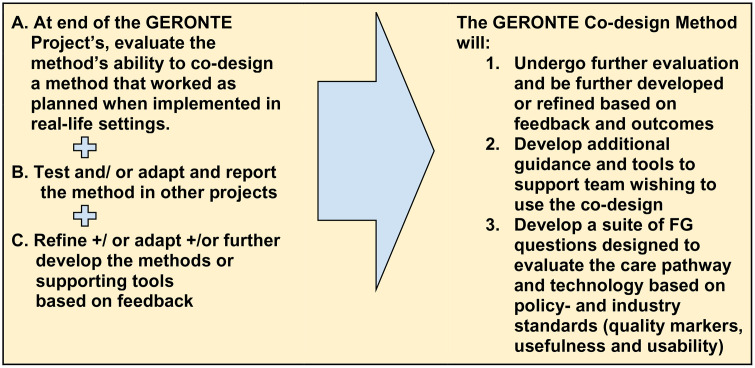
Overview of the process used to test PRA and FG co-design method following its use in GERONTE’s co-design.

The PRA and FG co-design method itself involved three design cycles consisting of an ‘ideation, early testing, and validation cycle’ (Fig 4).

The PRA and FG co-design method was tested and refined by using it in the GERONTE project. As such, we used each design cycle to gain feedback on the GERONTE PRA and FG Co-design method (Fig 3B).

#### Preparing, applying, and refining the method in GERONTE.

The first step involved preparing to apply the method, including:

- gaining ethical approval and
- recruiting and including participants.

The researcher team then conducted the co-design process in line with the GERONTE Co-design Protocol (S2 Appendix).

#### Number and type of participants and the nature of feedback to refine the method.

We had a total of 38 focus groups across 3 countries (Ireland, France, and Italy) with a total of 79 participants including clinicians, patient representatives, and managers (Table 2). We sought feedback on; whether the method achieved its aim overall, and within each design cycle; and, whether or not it was user-friendly. These feedback questions are presented in S1 Appendix.

**Table 2:** Summary of the number and categories of participants who gave feedback on the co-design method.

	Medical and other non-nursing professionals	Nursing	Patient & family member	Patient representative	Managers (service or IT)	Co-design researchers	Total no of participants
**Type and number of persons who participated in the co-design FG**	20, consisting of (medical 14, pharmacist 2, dietician 1, physio 2, occupational therapist 1	24	22 + 7	3	3	NA	79
**Type and number of persons who gave feedback on the co-design method**	17	16	4	2	2	11	52

The participants were experienced practitioners and service users who were considered able to identify if the co-design method was providing them with the opportunity to engage meaningfully and impact the intervention’s design. The number and experience of participants was; considered practical within the scope of the Randomised Controlled Trial of a complex intervention; and, met the FG and co-design methods literature recommendations (to ensure the number of participants per FG, involved multiple end-users, and enabled diverse discussion followed by consensus). The feedback, on the co-design method itself, from:

- the FG participants, was collected at the end of the FG or in a follow-up interview
- the research team, was collected during later GERONTE team meetings and/ or in written (email) format.

#### Evaluation and feedback used to refine the individual design cycles and the co-design method.

The FG participant and researcher feedback identified that the PRA and FG co-design method achieved the intended aims:

- to design and refine an integrated technology-supported care pathway
- while enabling continuous and meaningful engagement of different types of stakeholders in multiple locations.

We also sought feedback to identify if the individual design cycles achieved their intended aims. This feedback on the outcome of individual design cycles is identified in Table 3.

**Table 3:** Details of the feedback gained from each of GERONTE’s design cycles.

PRA & FG	Details of the information gained in this cycle	Outcomes from this cycle
**1st** **ideation cycle**	- Provided information, from multiple and different categories of end-users in different locations, on what they needed from an integrated care pathway - Identified the functions and user-friendly features the technology needed to have (again from multiple and different categories of end-users) - Enabled group discussion and agreement on the priority needs - Enabled rapid analysis, and sense-checking (through review and edit or approval by the FG participants) of the map of the care pathway and the technology’s functional and features.	- a map defining the care pathway (such as who needed to be involved and what information and support the professionals and patients needed) - an early prototype of the technology (identifying what functions the technology needed to perform).
**2nd** **User-testing cycle**	Enabled end-user to: - review, discuss, and feedback on the care pathway map - use, review, discuss, and feedback on the technology design (functions and features) prototype	- a refined map of care pathway (with agreement on the core health professional and data that should be included) - a refined prototype of the technology’s functions, content, and user-friendliness for the intended end-users (such as ensuring that key multimorbidity data was clearly presented to the professional, and sending only tailored information to the patient, and using larger font)
**3rd** **Validation + /- further refinement cycle**	Enabled end-user to: - review, discuss, and feedback on the updated care pathway map - use, review, discuss, and feedback on the updated technology prototype	- validation of the of care pathway map - validation of the technology and identification of additional features that would be helpful, but which were outside the scope of the RCT, but were noted for future post-trial adaptations.

#### Refinement made to the co-design method based on the feedback.

There was no change made to the use of:

- 3 cycles of FG and PRA as a way to co-design a technology-supported integrated care pathway.

The time between collecting the data (in the focus groups) and sending the findings to the participants for sense-checking was not reduced (as the rapid analysis and feedback was considered a key contributor to ensuring participant recall and robust research).

Thechanges *made to the method related to the practical aspects of conducting the FG*, such as:

- Allowing additional time for the ‘patient and family member’ interviews/ focus groups (to allow informal discussion and rapport building)
- Increasing the time interval between the co-design cycles (to add flexibility for the patients and clinicians and to allow the design team additional time to refine the design between cycles)
- the addition of individual interviews (using the comparable questions) for older adults who preferred one-to-one interaction [for Covid, personal-preferences, or time-related reasons] and for clinicians who were unable to attend the FG times.

### Fourth stage: Reporting and continued testing

The GERONTE clinical trial is ongoing. This co-design method will continue to be evaluated on its ability to co-design a ‘fit for purpose’ intervention (an integrated technology-supported care pathway) that meets end-users needs and context as part of a Randomised Controlled Trial. This co-design method will also be tested in other sites for its ability to co-design and/ or adapt an integrated technology-supported care pathway to different contexts.

*As the level of detail and regulation in policy- and industry-standards have progressed since this Project’s started, the fourth stage will also involve the development of an additional set of FG questions designed to gain end-users’ feedback on industry standardised measures of quality and user-friendliness*

Once the GERONTE project is complete and this method’s success or otherwise is known, this will be reported and submitted for further peer review and feedback.

## Results

This study resulted in the development, application, and refinement of the *Participatory Rapid Appraisal and Focus Groups* to Co-design Technology-supported Integrated Care.

The method was used in the GERONTE Project to co-design an integrated technology-supported care pathway. This method, Participatory Rapid Appraisal and Focus Groups in combination, enabled early, continuous, and meaningful involvement of multiple stakeholders across different locations and design iterations. It was found to be practical and user-friendly. The section will report the results of this study by identifying and explaining the method’s structure, steps, and output in detail.

The result of the ‘intervention’ (the integrated care pathway and the technology) will be reported separately as part of the GERONTE trial (where the effectiveness of the care pathway in improving older adults’ quality of care, experience, and life will be reported).

### Three cycles of participatory rapid appraisal and focus Group to co-design integrated technology-supported care

**Fig 5:**
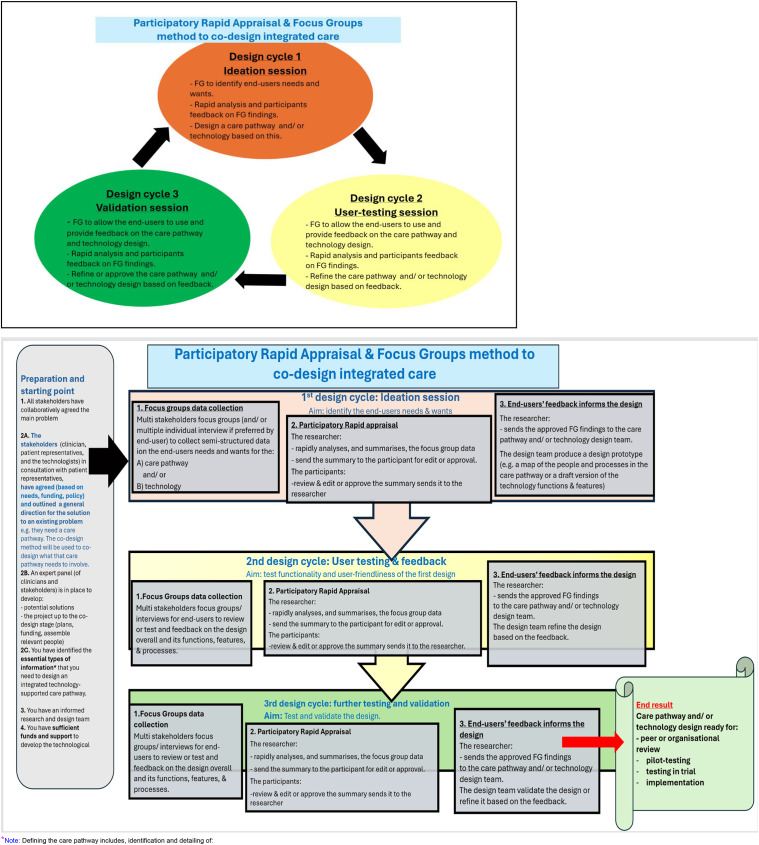
A: Overview of the 3 design cycles in the PRA and FG co-design method (and their aims). B: Detailed view of the steps and processes in PRA and FG to co-design integrated care. *Note: Defining the care pathway includes, identification and detailing of: which professionals need to be involved. what are the important times, events, and touchpoints along the patient's care journey (referral consultation, diagnostic testing, diagnosis consultation, treatment decision-making, treatment-specific milestones (such as 6 weeks post chemotherapy to assess tumour response). what information is needed, and when, by the health professional and patients.

The structure of the Participatory Rapid Appraisal and Focus Group method is **3 cycles of design, namely an: ideation, user-testing, and design validation** cycle. Fig 5A and Table 4 below provides an overview of each design cycle and thier aims. Fig 5B provides a detailed overview of the steps in the PRA and FG co-design method.

**Table 4:** Overview of the aims of each of the design cycles.

Design cycle	Design cycle and FG aim and questions
First cycle: ideation session	- identify participants’ needs and wants - develop an early design for the intervention (a map for the care pathway and a list of the functions and features needed from the technology).
Second cycle: user testing	- end-users review the care pathway and technology design/ prototype - end-users feedback on the intervention’s functionality and user-friendliness.
Third cycle: ‘validation of the design’	- end-users test and feedback **or** approve/ validate the design. *If the design is not considered complete* an additional cycle(s) of FG and PRA can be added until a validated design is reached.

*Each of the 3 design cycles involves*:

A. A number of multistakeholder ***focus groups*** from different sites to collect data on end-users’ individual and collective needs and priorities
B. ***rapid analysis (appraisal)****, and feedback to the FG participants for sense-checking,* of the FG findings (which sought end-user feedback on the design needs and priorities)
C. the ***creation*** or refinement ***of a design*** (for the care pathway and/ or technology).

### Structures and steps in the co-design cycle and method

The overall structure and sequence of the Participatory Rapid Appraisal and Focus Group method is outlined in Fig 5A and 5B. This is supported with a worked example in S2 Appendix (the GERONTE Co-design Protocol) which is provided in the Supplementary Materials. Fig 5A and 5B outline and describe:

- the preparation and starting point for the PRA and FG method
- the broad 3 steps in the PRA and FG method
- the data collection, analysis, checked (participant sense-checking), and synthesis within each step/ cycle
- how end-user feedback is used to design and refine the final intervention (integrated technology-supported care).

S2 Appendix (the GERONTE Co-design Protocol) provides a worked example of:

- the co-design aims
- PRA and FG method’s data collection, analysis, and synthesis methods
- FG participant inclusion and exclusion criteria
- The FG questions used in GERONTE (for the care pathway and FG)
- and detailed description of the role and responsibilities
- the timelines for the FG
- resources needed for the FG
- identification of factors that impact the time needed for the 3 design cycles

#### Note on Co-design preparation and starting point.

The PRA and FG to co-design integrated technology-supported care method is aimed at co-designing, as opposed to co-creation of integrated care. Co-creation is an end-to-end process. As such, the starting point for PRA and FG co-design method requires that there is agreement on the problems and scope of the project (agreement on the aims, potential solutions, budget, timelines, and/ or outcomes measures). For example, in the GERONTE project, the starting point was that there was agreement (based on empirical research, clinician and patient experiences, and expert and end-user experiences) that an integrated technology-supported pathway could improve the care, experiences, and outcomes. Though the overall aim is identified in advance of the co-design process, the inclusive nature of co-design *allows flexibility for the solution* to be adapted. *If the aim or potential solution are not agreed, then this co-design process would need to add an earlier step in which the problems, priorities, and perceived solution are agreed*.

### Adapting the co-design method to suit your needs

#### Focus groups or individual interviews.

Multistakeholder FG can be replaced or supplemented with individual interviews if preferred by some, or all, of the participants. The methodological difference would then be:

- potentially a need to shorten the interview time
- a need to analyse a higher number of transcripts/ interviews and create a higher number of interview finding reports for participant sense-checking
- A need to synthesise the data from across a greater number of interviews within each cycle

#### Care pathway design (FG) guides the technology design.

It is optimal to conduct the FG to design the care pathway and technology separately. The decision to collect the information on the care pathway and technology in the same or separate FG would depend on:

- the amount or complexity of the change that is needed to the existing care processes
- how well developed the technology is
- who is best suited to inform the design of the care pathway and the technology
- the participants’ knowledge, skills, and preferences (professionals with experienced in the area may have sufficient knowledge of the existing and intended, care process and technology, may be comfortable in identifying the co-design needs, while for example, newly diagnosed patients may not have sufficient familiarity with the treatment, care, or supportive care, processes, or of the potential role that technology can provide specific to their needs).

The implication for the co-design method is that, in practical terms the researcher will:

- need collaboration from service providers and users in identifying and including the range and type of stakeholders to include
- be organising, conducting, and reporting the care pathway and technology FG separately in most cases.

#### Questions within the FG.

In addition to the ideation, user-testing, and validation FG questions, additional questions can be added to some, or all, of the FG to gain feedback on policy- or industry standard measure related to the care pathway or technology. Such questions are likely to be specific to the category of end-user (e.g., patient, clinician, organisations) and are likely to be related to the ‘usefulness’ or ‘usability’ of the design.

## Strengths and limitations

A strength of this method is that it facilitates multi-stakeholder collaboration, which enables early and continuous collaboration in the design, *and/ or adaptation*, of an integrated technology-supported care. Additionally, the method’s use of rapid analysis enables time-bound project work. The inclusion of participants to sense-check the information (that will be used to inform the intervention design) enables rapid and efficient design refinement across the intervention’s design, implementation, or adaptation process. This supports real-life time and funding limitations and contextual differnces.

While this method requires further testing (of its ability to co-design an intervention that is successful and sustained when implemented), the use of empirically-based and familiar, practical methods, and the feedback and results in GERONTE, indicate that it may be an effective, straightforward, and user-friendly way to co-design interventions that involve multiple stakeholders.

While this method has been used to successfully co-design and refine an integrated technology-supported intervention, that is now being implemented on an RCT that is ongoing in a number of sites, a limitation is that it is not yet established if the co-designed intervention meets end users needs once implemented, though end-user feedback so far indicates it is useful and haining a positive impact on care services. Additionally further use and evaluation across different age groups and additional co-design projects (designing different interventions) is needed to evaluate its ability to co-design interventions meeting end-users’ needs and context. Work on reporting projects using this methodology continues.

## Discussion

This project has built on existing literature and experience and developed a novel replicable approach to co-design an integrated technology-supported care pathway.

### Current literature

Over the last three decades, there is a large body of co-design, IS, and related theoretical and empirical literature on their respective principles, practices, and definitions [Peters, 2024,33]. This has resulted in a comprehensive set of theoretical and practical guides for those undertaking co-design research [21,52,53]. The many local and national co-designed interventions that have succeeded when implemented demonstrate the utility and value of co-design principles and methods [54–60,61,62].

Most of these studies focus on the intervention (research output) or its impact. Many describe their co-design method, but, in line with the need to prioritise focus on research impact, the focus has been on the output, leaving lessspace to provide detailed description the method’s development, testing and refinement. Individually each of these studies have invested a lot of effort and time in developing a co-design method [57,63]. There are many methodological similarities across these methods, as they draw from the well-established co-design, and/ or IS, knowledge base [64]. The presence of a detailed robust and replicable method support research efficiency.

In real-world and time- and money-limited settings it is helpful to have a well-developed, structured and flexible, co-design method that maps onto the data collection, analysis, synthesis, and reporting processes [65,66]. The GERONTE project aimed to contribute to this gap, by developing and reporting a co-design method based in the existing evidence but applied to current healthcare needs (to implement integrated care).This co-design method provides a practical, replicable, evidenced-based approach which can be adapted to meet research needs and setting [67,68].

This project aimed to develop, test, and report in detail on a method that mapped PRA and FG onto the research process. In reporting this method, we considered the ease and accuracy with which this method could be re-used and how best to ensure it was connected in a clear way to its evidence base (PRA and FG) to enable users to easily recognise and draw from this base. We endeavoured to provide sufficient detail to make the method readily re-usable or adaptable by providing a full guide in the Appendices [69,70]. We also worked to situate this method within the existing literature by using existing common co-design and IS terminology [71,72,73].

### A method that aims to contribute to healthcare need

The current healthcare capacity, quality, efficiency, and policies identify the need for integrated care and services. A practical and efficient co-design method is needed to design the complex and interdependent changes needed to achieve integrated care.. The FG and PRA developed in GERONTE is presented as an evidenced-based, practical, flexible, affordable, replicable way to to identify and make change needed. Appreciably, there are limits, and consequently choices to what can be achieved within budget, time, or project limits, and we propose that PRA and FG can facilitates the collective discussion and agreement of these needs and priorities.. Consequently, while it is critical to have a co-design process giving meaningful inclusion and power-sharing [74], it is also necessary to have project leadership with relevant knowledge, alert to, balancing, identifying and developing all stakeholders priorities and needs within the co-design work [24].

### The method’s current place and further growth

Much co-design research is locally driven and time and cost-restrained [24,75], and as such there is benefit to having a defined, structured, evidenced-based method, that uses established discipline terminology, to co-design interventions. The FG and PRA method has drawn from and added to the incremental development of empirically-based replicable co-design method, by grounding its methods in the literature while aiming to address the practical need to have defined and detailed structures and processes that can be applied in other projects.

The use of PRA and FG in a structure way has been used with success in the GERONTE project, and while its ability to co-design an intervention (integrated technology-supported care pathway) that works as intended in the real world setting can only be determined at the completion and evaluation of the GERONTE RCT, the end-user feedback during the project has indicated:

- the intervention meets their needs
- the co-design PRA FG method enabled meaningful engagement across different end-users, locations, and designs and was user-friendly.

## Supporting information

S1 Appendix Method used to develop & test the PRA and FG Co-design integrated technology-supported care method. Appendix 1 provides a detailed description of the methods used to develop and test the PRA and FG method to co-design integrated technology-supported care in the GERONTE Project.
(DOCX)

S2 Appendix GERONTE Co-design Protocol. Appendix 2 provides the protocol used in the GERONTE project. It identifies the co-design aims and methods. It provides a worked example of: PRA and FG method’s data collection, analysis, and synthesis methods. FG participant inclusion and exclusion criteria. The FG questions used in GERONTE (for the care pathway and FG). And detailed description of the role and responsibilities. The timelines for the FG. Resources needed for the FG. Identification of factors that impact the time needed for the 3 design cycles.
(DOCX)
