# Supplementary material for: Participatory Rapid Appraisal and Focus Groups to co-design technology-supported integrated care
Source: PLoS One. 2026 Jun 16;21(6):e0299411. doi: 10.1371/journal.pone.0299411 (PMC13271751; doi:10.1371/journal.pone.0299411)
Supplement: S1 Appendix — Appendix 1 provides a detailed description of the methods used to develop and test the PRA and FG method to co-design integrated technology-supported care in the GERONTE Project. (DOCX) [file pone.0299411.s001.docx]

###### **Appendix 1: Record of the process used to develop and test Participatory Rapid Appraisal and Focus Groups to co-design Technology-supported Integrated Care as part of GERONTE**

The process used to develop, test, and refine *Participatory Rapid Appraisal and Focus Groups* to Co-design Integrated Care as part of the GERONTE Project* is described in detail to enable peer review, and/ or adaptation and re-use.

The GERONTE***** clinicians and management (oncologists and geriatricians, nurse specialist and the multidisciplinary team, patient representatives, and implementation team):

1. co-designed the GERONTE care pathway

2. collaborated with the technologists to develop and adapt technology to support that care pathway.

The implementation team researched, recorded, and reported the process.

The method was developed in four stages (S1A below). The four stages were:

1. Collaboration between the GERONTE stakeholders to identify the project’s co-design aims and needs (timelines, stakeholders involved, resources needed and available), and a review the literature to identify and propose a suitable co-design method
2. Stakeholder meeting to develop the co-design method and protocol
3. Applying, testing, refining, and reporting the method
4. Continued evaluation of the method (identifying the strength, weakness, and/ or its utility as a method to co-design other integrated care pathways or other complex intervention and/ or technology).

##### Supplementary 1A: Stages in the development of the GERONTE co-design method

| **Stage** | **Aim** | **Processes** | **Outputs** |
| --- | --- | --- | --- |
| 1 | Identify the co-design needs and a suitable method | **A.** Review of GERONTE documents and project team meetings to agree the co-design needs  **B.** Review of the literature to identify a suitable co-design method | Collaborative agreement that FG and PRA in combination met the co-design need |
| 2 | Develop the method and protocol  (identifying how FG and PRA would be used to co-design the GERONTE intervention) | **A**. Sequential multi-stakeholder meeting to define and detail how FG and PRA would be applied  **B.** Use the literature on co-design principles, PRA, and FG to inform the GERONTE co-design protocol | A co-design protocol that defined the co-design aims, data collection, analysis, synthesis, and reporting method and responsibilities |
| 3 | Applying and refining the method | The GERONTE co-design team:  **A.** applied the method in practice  **B**. refined the method (protocol) based on the research team and participants’ feedback | An integrated technology-supported care pathway (for older adults with cancer and other morbidities) |
| 4 | Reporting and continued testing | The GERONTE co-design team continues to:  **A.** evaluate the outputs from the method in the GERONTE project  **B**. refine the method and develop the reporting of the method based on feedback  **C.** identify suitable project to test the method | The early feedback on the method’s utility and user-friendliness identify it is a practical and useful method.  The reporting of the method has been refined based on peer review and feedback  The method’s ability to co-design an integrated technology-supported care pathway that is evidenced to be effective will be know at the end of the GERONTE RCT |

*****An EU-funded GERONTE project (GA945218) which aims to co-design, test, and prepare for EU-wide deployment an integrated technology-supported care pathway for older adults with cancer and other morbidities.

## First stage: Identify the co-design needs and a suitable method

The first stage (Figure S1), involved collaborative meetings to identify the project’s co-design aims and needs, followed by a review of the literature to identify a suitable co-design method.

##### Figure S1. Process used to identify a suitable co-design method

| **A. Review of project document**    **B. Project members meeting**  **to identify the co-design needs**    **C. Review of the literature to**  **identify a suitable co-design**  **method** |  | **Participatory Rapid Appraisal and Focus Groups in combination were identified as an empirically-based practical way to co-design an integrated technology-supported care pathway** |
| --- | --- | --- |
|  |  |  |
|  |  |  |

#### **Data sources and process used to identify the co-design needs**

The data sources used to identify the co-design aims, needs, and timelines were:

a) key GERONTE documents (EU-Grant Agreement and governing documents) that identified the outputs, timelines, and the research and reporting requirements

and

b) feedback from the project stakeholders and end-user (gained through collaborative meetings that identified individual and collective needs).

The data from the documents and meetings were analysed and synthesised thematically and discussed across subsequent meetings to agree collective and priority aims, needs, and timelines.

Agreement on the co-design aims and needs

The stakeholders agreed that GERONTE’s co-design aims and needs were to:

- co-design an integrated technology-supported care pathway that would improve care, outcomes, and experiences ([as defined by pre-defined objective clinical and health measurement], was economically viable [cost-benefit analysis to identify the total benefits as compared to the total costs], met end-users needs, and was sustainable, scalable, and adaptable [as determined by the end-user feedback and implementation evaluation)
- while ensuring early, continuous, and meaningful involvement of multiple stakeholders from different locations within a project’s time limits.

Agreement on the functions of a suitable co-design method

A co-design method was needed that:

- facilitated the involvement of, and discussion between, multiple stakeholders in different locations across a number of design iterations
- enabled rapid analysis and feedback from the researcher to the participants (to enable design refinement across design iterations)
- was sufficiently flexible to A) enable adaptation to stakeholders’ (such as patients, clinicians, managers) time schedules and B) to support stakeholders to introduce new information about their needs or contexts.

#### **Literature review to identify a suitable method**

A review of the literature identified FG and PRA in combination met GERONTE’s functional co-design needs. *FG provided an empirically-based practical way to collect semi-structured data identifying multiple stakeholders’ collective needs and priorities. PRA provided an empirically-based practical way to rapidly gain participant feedback to refine the intervention* (ensure continuous and multi-stakeholder involvement).

The data sources and process used werea multidisciplinary research panel who searched CINAHL, MEDLINE, SCOPUS and Google Scholar databases between 2010 and 2022 (limited to empirical research published in the English language), using the terms and string:

1. (‘Co-design’ OR ‘co-creation’ OR ‘co-production’ AND ‘methods’)

AND

1. (‘Implementation Science’ AND ‘theor*’ OR ‘model*’ OR ‘framework*)

AND

1. ‘healthcare’

to identify, analyse, and summarise the literature on co-design and Implementation Science principles and practices.

## Second stage: developing the method and protocol

The second stage (Figure S2), involved a sequence of multi-stakeholders meeting (between clinicians, research methodologists, patient representative groups, and managers) to:

- consider the suitability, strengths, and weaknesses of the proposed, and other, methods

- develop the co-design protocol to be used in the GERONTE’s project.

##### Figure S2. Overview of the process used to develop the co-design method and protocol

| **Multistakeholder meetings to:**  **A. Review and discuss the**  **literature identifying the**  **principles and practices of**  **co-design, FG, and PRA**    **B. Agree how to apply PRA and**  **FG in the GERONTE Project** |  | **1. Details of how PRA and FG can be combined (in 3 design cycles) to co-design a technology-supported integrated care pathway**  **2.GERONTE Co-design**  **Protocol developed** |
| --- | --- | --- |
|  |  |  |
|  |  |  |

#### **Co-design, FG, and PRA literature and multistakeholder meeting were used to develop the method**

Following agreement on the use of PRA and FG as a co-design method, a protocol was developed to:

1. document GERONTE’s co-design aims

2. define the participant recruitment and inclusion processes (for the FG), and

3. detail the practical tasks and processes required for the FG data collection and rapid analysis (PRA) and synthesis.

The data sources and processes used to develop the protocol included;

1. literature providing guidance on co-design, PRA, and FG principles and practices
2. sequential multi-stakeholder meeting (each with specific aims, outcome measures, and timelines) to develop, review, and refine the protocol across meetings (to ensure that it was robust, practical, reproducible, and achieved the intended aims).

(The GERONTE co-design protocol is presented in SUPPLEMENTARY 2 (Appendix 2) of this manuscript).

## Stage 3: applying and refining the method (in GERONTE)

The third stage involved applying and refining the method in GERONTE. It was an iterative process (Figure S3A and S3B), which is described briefly in the PLOS One manuscript, and presented in full detail in Appendix 1 (this attachment).

##### Figure S3A. Overview of the process used to apply and refine the co-design method

| **A. Apply the method**    **B. Gain feedback from**  **the researcher and**  **participants**    **C. Refine the method** |  | **GERONTE Co-design Method**  **developed**  **Report and send method for peer review, feedback, further development +/ or use in other projects** |
| --- | --- | --- |
|  |  |  |
|  |  |  |

##### Figure S3B. Detailed view of the three steps in applying and refining the co-design method.

| **Step1**  **Prepare** | **Step 2**  **Apply PRA and FG in GERONTE**  **Refine PRA and FG method based on feedback** | | **Step 3**  **Apply PRA and FG in GERONTE**  **Refine PRA & FG method based on feedback** | | **Step 4**  **Apply PRA and FG in GERONTE**  **Refine PRA & FG method based on feedback** | |
| --- | --- | --- | --- | --- | --- | --- |
| **Preparation**  **Gain ethics approval,**  **recruit, and include**  **participants** | **Apply the method as identified in the protocol.**  **Record the process.** | **Gain feedback from the FG participants and researchers on the method.**  **Use feedback to refine the method.** | **Apply the method.**  **Record the process.** | **Gain feedback from the FG participants and researchers on the method.**  **Use feedback to refine the method.** | **Apply the method.**  **Record the process.** | **Gain feedback from the FG participants and researchers on the method.**  **Use feedback to refine the method.** |
| How the method was applied and refined in GERONTE | **Conduct 1st**  **design cycle**  -FG and PRA (rapid feedback to participants within 48 hours of the FG) | **Feedback questions**  A. Did it identify your design needs?  B. What are its strengths, weaknesses, and outcomes?  C. Is it a user-friendly method?  Update the protocol in line with feedback and aims. | **Conduct 2nd**  **design cycle**  -FG and PRA (rapid feedback to participants within 48 hours of the FG) | **Feedback questions**  A. Did it identify your design needs?  B. What are its strengths, weaknesses, and outcomes?  C. Is it a user-friendly method?  Update the protocol in line with feedback and aims. | **Conduct 3rd**  **design cycle**  -FG and PRA (rapid feedback to participants within 48 hours of the FG) | **Feedback questions**  A. Did it identify your design needs?  B. What are its strengths, weaknesses, and outcomes?  C. Is it a user-friendly method?  Update the protocol in line with feedback and aims. |
|  | 14 FG |  | 14 FG |  | 10 FG |  |

#### **Preparing to apply the method in GERONTE.**

Ethical approval was gained at the individual sites. The researcher team then conducted the co-design process as described in the GERONTE Co-design Protocol (Appendix 2). In summary it identifies, the:

- co-design aim and timelines
- participant recruitment, selection, inclusion, and informed consent processes (FG participants included the clinicians, patients and/ or family members, and services managers involved in the provision of care to this patient groups)
- Practical details (tasks, responsibilities, and timelines) related to the FG and PRA data collection, analysis, synthesis, feedback, and reporting
- FG aim and questions
- co-design documentation, including the Participant Information Leaflet &consent forms.

Specific to the FG and PRA, it identifies in detail the tasks, responsibilities, and timelines for:

- the rapid analysis and feedback, within 48hrs (to the FG participants to sense-check the researcher’s completeness and accuracy in representing participants’ need and wants)

- the synthesis of data from the different FG and sites within each design cycle
- to provide synthesised feedback to the design team
- for the design team to develop draft design for the end-users to review and refine at the next cycles of FG.

#### **Including participants and starting the co-design process in GERONTE**

The researchers recruited, selected, and included participants in line with the inclusion and selection criteria (participants to represent all types of end-users). The oncologists (PS and HW) and Geriatrician (MH, SOH, SR, and NS), and the nurse specialists working at the associated clinical sites (led by CK), led the care pathway co-design. A collaboration between the clinician, technologists, patient representative organisation (ESE) and implementation team, supported the design and refinement of the technology.

#### **Applying the co-design method**

The researcher team conducted the co-design process in line with the GERONTE CO-design protocol (Appendix 2).

In summary the *co-design method itself involves three (3) design cycles consisting of an ideation, early testing, and validation cycle of the intervention*.

The application and *refinement of the method also involved three (3) cycles of ‘application, feedback, recording, and refinement*’ (Figure S3B). In practical terms, each design cycle was used to gain feedback on and refine the co-design method (to identify what worked well and what we needed to change).

The implementation team recorded the co-design process, noting:

- fidelity or adaptations to the process

- participant experiences and feedback on the method

- what went as intended and what did not

- the outputs from the individual design cycles and from the overall process.

#### **Type of participants and the nature of feedback**

The number (N38) of co-design FG is identified in Figure S3B. The number (N79) and category of participants involved in the FG and who provided feedback (N52) on the method itself are identified in Table S1 below.

The participants were informed and experienced service users considered able to identify if the co-design method was providing them with the opportunity to engage meaningfully and impact the process. The number and experience of participants was considered practical within the scope of the Randomised Controlled Trial of a complex intervention.

The feedback from:

- FG participants was collected at the end of the FG or in a follow-up interview
- The research team was collected during subsequent GERONTE team meetings and/ or in written (email) format.

#### **Table S2: Number and types of participants that gave feedback on the method**

|  | **Medical**  **And other disciplines** | **Nursing** | **Patient and family member** | **Patient representative body** | **Managers**  **(hospital services or IT)** | **Researchers** | **Total no of participants** |
| --- | --- | --- | --- | --- | --- | --- | --- |
| **Type and number of persons who participated in the FG** | 20, consisting of:  -medical 14, pharmacist 2,  dietician 1, physio 2, occupational therapist 1 | 24 | 22 + 7 | 2 | 3 | NA | 79 |
| **Type and number of persons who gave feedback on the co-design method** | 17 | 16 | 4 | 2 | 2 | 11 | 52 |

#### **Recording the outcomes to determine if the individual design cycles and the co-design method achieve their aims**

The new co-design method achieved the intended aims:

- to design and refinement of an integrated technology-supported care pathway**

- while enabling continuous and meaningful engagement of different types of stakeholders in multiple locations.

In order to refine and improve the method, we also considered the outcomes of the individual design cycles.

##### Table S2: Details of the feedback gained from each of GERONTE’s design cycles

| **PRA & FG** | **Information gained from participants** | **Design results** |
| --- | --- | --- |
| 1st ideation cycle | - provided information, from multiple and different categories of end-users in different locations, on what they needed from an integrated care pathway  - identified the functions and user-friendly features the technology needed to have (again  from multiple and different categories of end-users)  - enabled group discussion and agreement on the priority needs and  - enabled rapid analysis, and sense-checking (review and edit or approval by the FG participants) of the care pathway map and the technology’s functional and features. | The first design cycles resulted in the development of;  - of a map defining the care pathway (such as who needed to be involved and what information and support the professionals and patients needed)  - an early prototype of the technology (identifying what functions the technology needed to perform). |
| 2nd  User-testing cycle | Enabled end-user to:  - review, discuss, and feedback on the care pathway map  - use, review, discuss, and feedback on the technology prototype | The first design cycles resulted:  - in the refinement of care pathway map (such as the agreement of the core health professional and data  - refinement of the technology’s functions, content, and user-friendliness (such as ensuring that key multimorbidity data was clearly presented to the professional, sending only tailored information to the patient, and using larger font) |
| 3rd  Validation +/ - further refinement | Enabled end-user to:  - review, discuss, and feedback on the updated care pathway map  - use, review, discuss, and feedback on the updated technology prototype | The third design cycles resulted:  - validation of the of care pathway map  - validation of the technology and (identification of additional features that would be helpful, but which were outside the scope of the RCT, but were noted for future post-trial adaptations). |

#### **Changes made to the method**

There was no change made to the use of:

- 3 cycles of FG and PRA as a way to co-design an integrated care pathway.

The main changes made to the method related to the practical aspects of the FG, such as:

- the duration of time for the ‘patient and family member’ interviews/ focus groups (which was increased to allow informal discussion and rapport building)
- the intervals between the co-design cycles (which was increased to add flexibility for patient and clinician participants’ and to allow the design team time to refine the intervention design between cycles)
- the addition of individual interviews (using the comparable questions) for some older adults who preferred one-to-one interaction [for Covid, personal-preferences, or time-related reasons] and for clinicians unable to meet at the FG times.

The time between collecting the data (in the focus groups) and sending the findings to the participants for sense-checking was not reduced (as the rapid analysis and feedback was considered a key contributor to ensuring participant recall and robust research).

####

#### **Summary of the ‘refinement questions and feedback’**

A summary of the questions seeking feedback on the method are provided below.

The ‘review and refinement questions’ were purposely direct and open in order to gain understanding, enable replicability, and to minimise the burden on respondents.

The questions focused on whether or not:

1. ‘the co-design method had identified their needs and priorities
2. ‘the co-design method had resulted in a design that appeared to meet their needs’
3. ‘the co-design was a comfortable, practical, and user-friendly experience’
4. ‘they had any other feedback or suggestions’ (from both the intervention end-users and co-design team about the co-design process).

The feedback identified that the new method:

1. was successful in focussing the co-design process around, and identifying and prioritising, their needs
2. had resulted in a design that appeared to meet their needs
3. enabled collaboration and feedback across multiple stakeholders working within busy clinical and trial settings
4. enabled rapid and progressive feedback design iterations
5. w[as comfortable, practical, and flexible

Additional feedback was that;

1. while for many of the clinicians and professionals (end users) focus group format was feasible, useful, and preferable
2. when clinicians were severely time constrained, they preferred individual interviews (over focus groups)

older or more frail adults demonstrated a preference for individual interviews over interdisciplinary or multi-patient interviews, expressing a wish to contribute (and provide information) but preferring the flexibility and shorter duration facilitated by individual interviews.

## Stage 4: Reporting and continued testing

The GERONTE clinical trial is ongoing. This co-design method will continue to be evaluated on its ability to co-design a ‘fit for purpose’ intervention (an integrated technology-supported care pathway) that meets end-users needs and context as part of a Randomised Controlled Trial. This co-design method will also be tested in other sites for its ability to co-design and/ or adapt an integrated technology-supported care pathway to different contexts. Once the GERONTE project is complete and this method's success or otherwise is known, this will be reported and submitted for further peer review and feedback.

##### Figure S4. Overview of the process used to test and refine the PRA and FG co-design method following its use in GERONTE’s co-design

| **A. At end of the GERONTE**  **Project’s, evaluate the**  **method’s ability to co-design**  **a method that worked as**  **planned when implemented in**  **real-life settings.**    **B. Test and/ or adapt and report**  **the method in other projects**    **C. Refine +/ or adapt +/or further**  **develop the methods or**  **supporting tools**  **based on feedback** |  | **The GERONTE Co-design Method will:**   1. **Undergo further evaluation and be further developed or refined based on feedback and outcomes** 2. **Develop additional guidance and tools to support team wishing to use the co-design** 3. **Develop a suite of FG questions designed to evaluate the care pathway and technology based on policy- and industry standards (quality markers, usefulness and usability)** |
| --- | --- | --- |
|  |  |  |
|  |  |  |
