# Supplementary material for: Participatory Rapid Appraisal and Focus Groups to co-design technology-supported integrated care
Source: PLoS One. 2026 Jun 16;21(6):e0299411. doi: 10.1371/journal.pone.0299411 (PMC13271751; doi:10.1371/journal.pone.0299411)
Supplement: S2 Appendix — Appendix 2 provides the protocol used in the GERONTE project. It identifies the co-design aims and methods. It provides a worked example of: PRA and FG method’s data collection, analysis, and synthesis methods. FG participant inclusion and exclusion criteria. The FG questions used in GERONTE (for the care pathway and FG). And detailed description of the role and responsibilities. The timelines for the FG. Resources needed for the FG. Identification of factors that impact the time needed for the 3 design cycles. (DOCX) [file pone.0299411.s002.docx]

###### Appendix 2: GERONTE Co-design Protocol and Guide

This protocol details the application of Participatory Rapid Appraisal and Focus Groups co-design method in GERONTE Project (Horizon 2020 Grant approval 945218. Note, identifying terms redacted in line with agreements).

### The development and purpose of this protocol and guide

The primary aim of this guide is to provide the practical detail on how Participatory Rapid Appraisal (PRA) in combination with Focus Groups (FG) can be used to co-design and/ or adapt the GERONTE* intervention during and after the clinical trial. It will also support robust and consistent data collection, analysis, synthesis, and reporting processes across the GERONTE trial sites.

*GERONTE is funded under EU Horizon GA 945218

The process described in this guide is designed:

- based on the co-design, PRA, and FG literature
- to meet GERONTE’s co-design needs
- based on the practical considerations of using PRA and FG to co-design an integrated technology-supported care pathway.

The co-design needs were identified and agreed:

1. by reviewing the GERONTE Grant Agreement and other guidance documents, such as A) documents from the formal groups (Work Package) responsible for defining the care pathway and technology [Diak, Bordeaux, and University College Dublin University, and the technology company], and B) Ethical approval documents
2. across collaborative meetings between GERONTE clinicians, methodologists, and managers to identify the aims, timelines, outputs, and the research and reporting requirements.

The theoretical and practical consideration needed to apply PRA and FG to co-design an integrated technology-supported care pathway were identified by:

1. reviewing the literature and adhering to co-design, PRA, and FG principles and literature on implementing complex interventions and technology into healthcare
2. across collaborative meetings between GERONTE clinicians, methodologists, and managers to identify and agree on the practical needs.

### Using this guide across multiple trial sites

The guide is intended for use by the GERONTE co-design and implementation teams.

The benefit of a consistent and collaborative approach is that it:

- maximises the data collection efficiency and effectiveness across FG, sites, and design iterations
- minimises the research burden on participants (as efficient research)
- ensure the scientific value of GERONTE results (through consistent and replicable co-design)
- develops a robust reproducible method to implement and/ or adapt GERONTE at additional sites posttrial (in line with trial results).

### 3. The GERONTE intervention and its aims

The GERONTE project’s overall aim is to improve the quality of life for older multimorbid patients, while reducing the overall costs of care by designing, testing, and preparing for EU-wide deployment an integrated technology-supported care pathway. The pre-determined objectives of the care pathway and technology are set out in the GERONTE Grant Agreement (GA) and they impact the scope and direction of the interventions design. In summary, *the care pathway needs to identify, structure, map, and detail the core professionals, information, and processes needed to provide patient-centred holistic care*. The technology needs to *provide functions, such as collection, storage, sharing, and analysis of health data,* to support this care pathway.

In practical terms this means the technology needs to:

- enable the coordination of care (between the different disciplines and health services)

- enable the provision of support to patients across the different stages of their cancer journey (including the timely registration of symptoms and patient-reported outcomes)

- provide different and independent functions to different end-user

- provide different and independent, and some shared views, to the different end-user

- ideally have capacity for interoperability with existing local technology.

The detailed specification of what the care pathway and technology needs to achieve are identified in the GA.

### 4. GERONTE’s co-design aims and approach

The co-design method:

- aims to provide information on the end-users needs and wants from the care pathway and/ or technology
- can be used post-trial to support pre-implementation and/ or adaptation to additional sites

The questions in the FG will be developed to gather feedback on:

- what participants want and need from the care pathway and technology
- on their ICT use
- on existing care pathways.

GERONTE’s approach to designing the integrated technology-supported intervention involves *3 cycles of design: an ideation, user-testing and feedback, and design validation session.*

***Figure A (below)*** provides an overview of the design cycles and the core work and outputs from each to support the co-design team to plan and conduct.

### 5. Overview of the co-design process for GERONTE

**GERONTE’s co-design process has 3 design cycles.**

Each design cycle involves Participatory ***Rapid Appraisal*** of ***data collected*** in the ***Focus Groups*** (there will be multiple FG in each cycle as GERONTE has multiple sites).

The co-design process identifies some of the ***critical preparation needed***, including agreement of the problem and an understanding of the essential safety, policy, funding, or feasibility considerations (to ensure that the new intervention can be safe and materialise). This understanding does not preclude end-users taking the FG discussion in any direction, but it will act to inform the design output (which will then be considered by the same end-users).

The FG, ideally with a mixture of different categories of stakeholders, collect semi-structured information on the end-users’ individual and collective needs and priorities.

The data from each FG is analysed rapidly by the researcher, and their summary sent back to the FG participants for sense-checking (for verification or editing).

The verified FG data from the different sites is sent to the design team.

The output from the design team is reviewed at the next FG and the feedback collected, analysed and sense-checked and synthesised as above and sent back to the design team to refine the intervention.

In GERONTE, there will be *two streams of FG within each cycle, one for the care pathway and one for the technology*. Feedback from the two designs can be reviewed by the participants during the focus groups to confirm their compatibility and completeness.


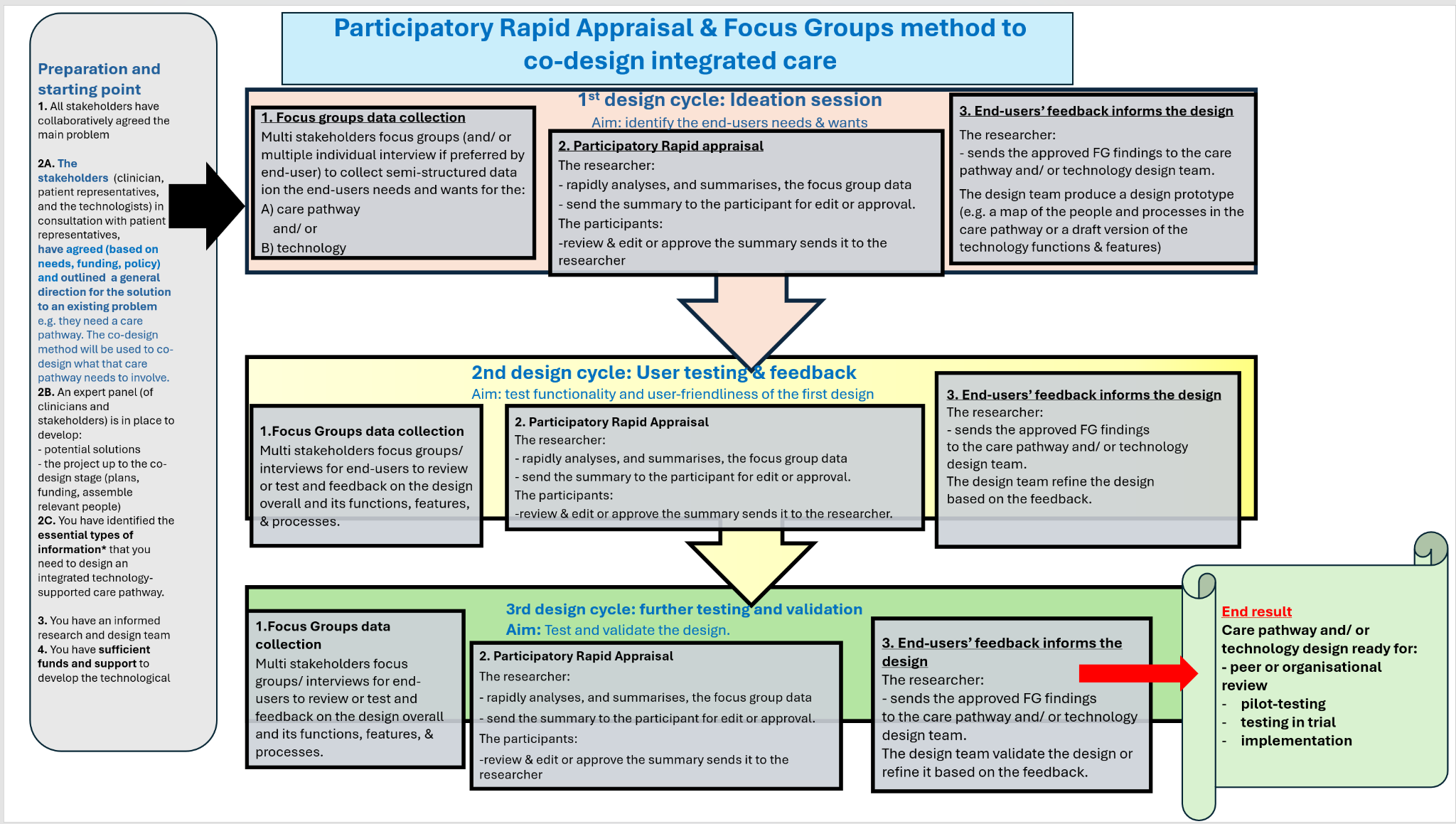


#### **Figure A: Process and steps in PRA and FG to co-design integrated care method**

####

### 6. The aim and question within each cycle

In GERONTE, the care pathway FG and technology FG were conducted separately to:

1. allow the core structure and people of the **care pathway to be identified and to guide the design of the technology** (as the technology’s functions depend on the people and processes and what functions and features support their needs and workflow)
2. ensure sufficient time with each FG for participants to consider and provide information on both the care pathway and technology
3. minimise the time burden on the participants (patients who'd recovered from cancer and were older adults and clinicians who were on tight time schedules).

###### Grid 1. Overview of the aim and questions within each cycle

| **Design cycle aim** | **Cycle and FG questions** |
| --- | --- |
| First cycle, ideation cycle | The ideation cycle, and FG questions, aim is to:  - identify participants’ needs and wants  - develop an early design for the intervention (a map for the care pathway and a list of the functions and features needed from the technology). |
| The second, design testing | The second, design testing, cycle’s aim is to provide participants with an opportunity:  - to review the care pathway and technology design/ prototype  - to feedback on the intervention’s functionality and user-friendliness. |
| The third, ‘further testing and feedback’ or ‘validation’ cycle | The third, further testing and feedback or validation, cycle’s aim is to:  - facilitate further testing and feedback.  If the design is not considered complete an additional round(s) of FG and PRA can be added until a design is reached. |

**7. Proposed timeline for the design cycles**

Figure B below provides an overview of the co-design timeline and design cycles. This timeline is dependent on a number of factors or assumptions, which are identified and considered below.

###### Figure B: Example timeline and sequence for PRA and FG method

|  | **Month 1** | **Month 2** | **Month 3-4** | | **Month 4-5** | |
| --- | --- | --- | --- | --- | --- | --- |
| **Care**  **Pathway**  **design** | **1st design cycle**  **Ideation** | | **2nd design cycle**  **User testing and feedback** | | **3rd design cycle**  **Testing & feedback or design validation** | |
|  | **FG** to develop a:  - draft definition of the care pathway.  **Rapid appraisal** to ensure participants views are reflected in this definition | The clinician and patient representatives:  - define, detail, and **map the care pathway*** | FG for the end-users to **discuss and give feedback on the:**  **- care pathway map and details** | The clinician and patients:  - develop and **refine the care pathway map** based on the feedback received | FG to:  -review, test, and give further feedback or validate the care pathway map and details | Validated design for the care pathway |
| **Technology**  **design** | **1st design cycle**  **Ideation** | | **2nd design cycle**  **User testing and feedback** | | **3rd design cycle**  **Testing + feedback or design validation** | |
|  |  | FG and PRA to **identify the function and feature that the technology needs** to perform to support the care pathway.  The technologist, in collaboration with the clinicians and patient representatives, develop an early prototype of the technology for testing | FG for the end-users to;  **- test and give feedback on the technology’s functions, features, and user-friendliness** | The technologist, in collaboration with the clinicians and patient representatives, **develop and refine the technology** in line with the feedback received | FG to:  -review, test, and give further feedback or validate the technology | Validated design for the technology |

*Defining the care pathway includes, identification and detailing of:

- which professionals need to be involved
- what are the important times, events, and touchpoints along the patient’s care journey (referral consultation, diagnostic testing, diagnosis consultation, treatment decision-making, treatment-specific milestones (such as 6 weeks post chemotherapy to assess tumour response),
- what information is needed, and when, by the health professional and patients.

### 8. Recruiting participants for the co-design FG

The recruitment process at each co-design host site is guided by the:

- the FG aim, and the participant inclusion and exclusion criteria (see Grid 2 below which lists the inclusion & exclusion criteria)
- the ethical approval guidelines for each host site
- Practical consideration in accessing the type and number of participants needed.

As a practical guide, the usual recruitment process is:

1. Identifying a site where healthy seniors patients (as a priority), patients, informal caregivers and the relevant Health Professionals (HP) can be accessed.

2. Contacting relevant stakeholders to propose recruitment

3. Gaining the relevant ethical approval

4. Advertising & recruiting for the study

5. Follow-up with potential participant (determine eligibility, arrange and support participation in the FG)

6. Maintain relevant records securely to enable contact. Ensure data saved and deleted in line with the relevant ethical approval.

### 9. Categories and number of participants

The FG require four (4) categories of participants (Grid 2 below):

1. Healthy Seniors (as a priority)

2. Patients

3. Informal caregivers

4. Health Professionals (HP) of which there are (an additional) 4 sub-categories.

a. Healthcare team for cancer (Oncologist)

b. Healthcare team for comorbidities (other Medical specialist)

c. Nurses (as defined by the local health system),

d. Other HP (including Nutritionist, Pharmacists, Ambulance Personnel, Physiotherapist)

Each research site will identify recruitment sites for the different types of participants. Clinical sites will be used for HP and as a guide, Voluntary Senior Organisation, National or local, statutory or voluntary, cancer organisations and Cancer centres are potentially suitable organisations to approach for patient representatives.

In calculating the number of participants to recruit, consideration is needed on:

1. how many participants will be needed to gain a comprehensive overview on the what the care pathway and technology design needs and wants are
2. how much time each participant can give (such as older adults post cancer treatment)
3. how much experience or how much consideration each participant has given to the idea of an integrated technology- supported care pathway.

In practical terms:

1. an estimate of how many to include will need to be made
2. then a small number of participants included, and
3. more participants included if and as needed (there is no clear agreement on what the care pathway or technology needs to do).

In line with policy, healthy seniors are a priority for the co-design process.

In general, for each of the categories of participants (healthy seniors, patients, informal carers and health professionals), the research site will have 3 focus groups, and 3 interviews, with each category.

###

## Grid 2. Participant inclusion and exclusion criteria.

| **Participant inclusion and exclusion criteria** | | |
| --- | --- | --- |
| Patients | Caregivers | Health professional |
| **Inclusion Criteria:**  Age ≥ 70 years  -Non-frail or Pre-frail condition  - First stages of Cancer (1-2) or in complete remission or in partial remission for at least 6 months  - Cancer types targeted in the overall project: Lung, Prostate, Breast, Colorectal  - Multimorbid: defined as cancer plus at least one severe morbidity  - Technology Acceptance    **Exclusion Criteria**  - Cognitive decline  - Clinically unstable patients in the clinical judgment of the investigator  - -Terminal illness (life expectancy < 12 months)  - Patients with a sensory deficiency or with a certain level of motor disability that, according to GERONTE partners, precludes the possibility of interacting with GERONTE  - Subjects unwilling or unable to provide consent or unable to participate safely in the intervention program.  Patients with acute or uncontrolled psychiatric disease | **Inclusion Criteria:**  Age ≥ 18  - Non-frail or Pre-frail condition  - Technology Acceptance  - Caregiver to an older patient with Cancer and at least one comorbidity, regardless if patient is involved in the project or not    **Exclusion Criteria:**  -Subjects unwilling or unable to provide consent or unable to participate safely in the intervention program.  -Caregivers with a sensory deficiency or with a certain level of motor disability that, according to GERONTE partners, precludes the possibility of interacting with GERONTE. | **Inclusion Criteria:**  Technology Acceptance  - Practising Health Professional dealing with patients with Cancer with comorbidities according to a list determined in WP1  - Practising in the healthcare centres identified and involved in the project    **Exclusion Criteria:**  - Subjects unwilling or unable to provide consent or unable to participate safely in the intervention program. |

###

### 10. Resources needed to conduct the FG

###### Grid 3. The following resources will be required to conduct the FG

| Personnel resources | Physical resources  In-person FG/ interview | Physical resources  Online FG/ interview |
| --- | --- | --- |
| Project coordinator  Moderator  Facilitator/  Notetaker  Observer (optional) | Data collection equipment: Audio recording device x 2, stationary to take own notes, individually packed stationary for participants to make notes during discussion.  Booked venue (physically accessible and convenient for participants, private, quiet, adequate temperature, lightening, seating and space, compliant with health regulations).    2^nd^ and subsequent FG/ interviews. Access to GERONTE app via a physical device or web-based access.  Refreshment (snack food/ hydration with consideration for participants preferences or dietary requirements)  Infection control consideration: Relevant hand hygiene sanitiser, personal protective equipment (face masks or visors), and waste disposal equipment.  Data security equipment: method to ensure data collection devices security in transport. | Data collection equipment: Audio-recording device that enables secure recording in line with the relevant ethical guidelines. Stationary to take notes.  Online: liaise with participants prior to FG to ensure access to technological devices, software that is compatible with the online teleconferencing method (zoom, google meets, Microsoft teams), and internet connection.  2^nd^ and subsequent interviews/ FG. Access to GERONTE app via a physical device or web-based access. |

###

###

### 12. Practical preparation to conduct the FG

###### Grid 4 ‘A guide to the ‘on the day’ checklist for the FG is provided below.

| **Overview of the on-the-day preparation** |
| --- |
| **Facilitator:**  In-person FG: Arrives early and undertakes checks of the room and equipment.  Is available to respond to participant requests for support (locating/ accessing the FG).  If online: contact the relevant participants to provide support in gaining access to online meeting.  Liaise with the moderator to ensure shared understanding of the routine and responsibilities.  Set up and sound check recording device(s).  Set up devices technology to present PowerPoint  Use PowerPoint presentation to ensure a clear introduction  Confirms and collects written consent, facilitates consent process where relevant.  Greets and (if in-person) directs participants to seating (in U-shaped or semi-circle format). Covid related guidance on proximity (2m apart). Identifies availability and location of refreshments  Assumes own position in the room. Confirms to the moderator that the recording device(s) are being commenced. Begins note-taking once FG begins.  Ceases recording as directed once Moderator has closed the FG.  Support moderator in securing the data collected and other resources.  Completes any necessary administration in relation to the booked venue.  Allocates 1 hour to go through notes, and makes additional notes, connections in conjunction with the moderator (see Grid 7b for more details)    **Moderator:**  Arrives early to collaborate and support the facilitator as needed.  Revises the aim, routine, timing, and questions in the FG.  Greets participants and builds trusting rapport using general conversation relevant to the participants’ background.  Introduction and moderation of FG as described in Appendix 2 and Grid 7a (below) |

######

###### Grid 5. Suggested structure and duration of a FG

|  | **Suggested structure of the FG** | **Duration** | |
| --- | --- | --- | --- |
|  |  | **Health**  **professional FG** | **Older adult or**  **caregiver FG** |
| 1 | Informal introduction and greeting.  Formal check/ confirmation of consent to undergo and record the FG | 5 | 10 |
| 2 | Commence recording.  Formal introduction of project, FG aim and approach/ rules for the FG using standard PowerPoint presentation.  Invite for question regarding the FG aim, process and follow up. | 5 | 5 |
| 3 | Care pathway question  Technology questions | 30  40 | 20  20 |
| 4 | Summary of the FG’s aim, main points of discussion raised.  Invite adjustment to summary or any relevant points not addressed  Thanks participants and close FG.  Secure data collected and any consent.  Support participants as needed. | 10 | 10 |
|  | Overall time of the FG | 90 | 65 |
| 5 | Allocate time to sit with facilitator to review and add to notes as needed | 50 | 50 |

### 13. Suggested care pathway and technology questions

The Moderator guides the discussion based on the FG aims and questions. The Facilitator takes relevant notes, such as whether there was overall agreement/ disagreement among participants, and checks for non-verbal confirmatory or opposing cues during the discussion. Each co-design FG can develop their own and shared topic guide and questions based on the core topics around:

- What are the participants’ needs and wants around the care pathway
- What are the participants' needs and wants around the technology?

Grid 8a and 8b below contains sample questions.

### 14. Data collection, analysis and reporting

No Personal Data (as defined by the 2018 General Data Protection Regulations [GDPR]) will be collected during the FG. Data from the FG/ interviews will be audio recorded.

Audio recording will be transcribed within 24 hours of the FG. Participant personal identity will be anonymised (if Personal Data has been inadvertently disclosed by participants) at the transcription stage.

Transcription can be undertaken using manual or an automated transcription service. If using an automated transcription service, the host must verify to the GERONTE Project Coordinator that the transcription service is accurate and meets the relevant ethical approval, and privacy and confidentiality requirements.

The Facilitator’s notes, taken during, and in the debrief immediately after the FG, will be added to the transcript notes.

A ‘Participatory Rapid Appraisal’ (PRA) method is recommended for analysis and reporting of the FG as it:

- ensures robust findings through member-checking

- will enable feedback to the software developers or care pathway team in a timeframe consistent with their needs.

- is consistent with the project’s co-design approach

DCU will provide training and support for the people doing the rapid reporting to ensure a common approach. The overall structure of PRA and FG method and details of how to apply it are presented in Figure A (above) and Grid 6 (below)

## Grid 6: Aims of the design cycles

| **Design cycle** | **Design cycle and FG aim and questions** |
| --- | --- |
| First cycle:  ideation session | - identify participants’ needs and wants  - develop an early design for the intervention (a map for the care pathway and a list of the functions and features needed from the technology). |
| Second cycle: user testing | - end-users review the care pathway and technology design/ prototype  - end-users feedback on the intervention’s functionality and user-friendliness. |
| Third cycle:  ‘validation of the design’ | - end-users test and feedback **or** approve/ validate the design.  *If the design is not considered complete* an additional cycle(s) of FG and PRA can be added until a validated design is reached. |

.

## Grid 7a. Moderator’s role, key reminders

| **Role** | **Details** | |
| --- | --- | --- |
| **Moderator role** | The moderator's role is to, in an impartial manner, facilitate inclusive, meaningful, and fluid discussion focussed on the FG topic and aim. . In a neutral and professional manner, they will ensure the discussion and time is allocated fairly and moderate as necessary by reframing if and as necessary. | |
| **Preparation** | Preparation  Knowledge of the project & focus group.  Knowledge of the health care system and cancer journey (though will not use to prompt discussion). Be alert and free from distractions. Discipline and active listening. Familiar with questioning route    Introduction  Purposeful small talk to create friendly and trusting environment  Observant of participants and seating  Make a clear, smooth, snappy (standardised) introduction    Have a stock of appropriate useful phrases to develop discussion.  That’s interesting,…  …can you tell me more about that  … can you give me an example | Control reactions to participants  Verbal and non-verbal  Head nodding  Uses short verbal responses.    Use subtle group control.  Experts, Dominant talkers, Shy participants, Extended discussion with minimal new content.  Interjects in reassuring manner and directs the conversation to the groups or other person with an open exploratory question.  Uses appropriate conclusion. 3 step conclusion  (1.Summarise with confirmation, 2.Revise purpose and ask if anything has been missed, 3. Thanks participants and bids farewell). |
| **Key reminders** | FG time allocation  HP: 1 to 1.5 hours overall  Patients, informal caregivers and healthy seniors    Breakdown time plan  Grid 5  Greeting & introduction: 10 minutes    Questions: 100 or 70 minutes. | 10 broad questions each with or 9 3 follow on questions as necessary.    Summary, conclusion & farewell: 10 minutes  Moderator will need to be available for conversation with participant afterwards (Ensure book additional time in venue). |

##

## Grid 7b. Facilitator’s role, key reminders

| **Role** | **Details** |
| --- | --- |
| **Facilitator** | The main function of the facilitator is to ensure that a meeting runs smoothly and achieves the desired objectives. This will require preparation and multi-tasking.  He or she is a mediator, ensuring communication between all the participants. To put them at ease and to ensure that the event runs smoothly, the facilitator also plays the role of architect. He or she is responsible for arranging the meeting room, preparing the table, adjusting the room's lighting, deciding where each participant should sit, etc. |
| **Preparation** | Facilitator:  Arrive early and undertake checks of the room and equipment.  Is available to respond to participant requests for support in locating or accessing the FG or setting up technological devices.  Liaise with the moderator to ensure shared understanding of the routine and responsibilities.  Set up and sound check recording device(s)  Set up devices technology to present PowerPoint  Use PowerPoint presentation to ensure a clear introduction  Confirms and collects written consent, facilitates consent process where relevant  Greets and directs participants to seating (in U-shaped or semi-circle format). Covid related guidance on proximity (2m apart). Identifies availability and location of refreshments.  Assumes own position in the room. Confirms to the moderator that the recording device(s) are being commenced. Begins note-taking once FG begins.  Ceases recording as directed once Moderator has closed the FG.  Support moderator in securing the data collected and other resources.  Completes any necessary administration in relation to the booked venue.  Allocates 1 hour to go through notes, and makes additional notes, connections in conjunction with the moderator. |

### 15. Roles and Responsibilities for Partners

E-Seniors are responsible for the coordination of the co-design FG sessions.

Each host Partners is responsible for: contribution to collaboration, and for the planning (including collaboration with Partners for shared FG), organising, conduction, and reporting from their own FG/ interviews.

**Data security**

Each Partner is responsible for the management, security and privacy of all data they receive. The person responsible for data collection, analysis and reporting will ensure that the data is managed (collected, analysed, anonymised, saved, stored, reported, shared, and deleted) in line with 2018 General Data Protection Regulation requirements and as stipulated by their ethical approval agreement.

**Translation**

Translation (from one language to another) is the responsibility of the host partner. If using external translation service, the host must verify to the GERONTE Project Coordinator that the service is accurate and meets the relevant ethical approval, and privacy and confidentiality requirements prior to its use.

**Communication responsibilities around FG feedback**

The host Partner will communicate the ‘FG/ interview report’ in English to all Partners ideally within 2 working days of the FG taking place.

The host Partner will communicate the ‘summative report ‘ in English or French, at the end of the week, or as otherwise pre-agreed between GERONTE software developer Company and the host site. The host Partner will copy all Partners in this communication. The summative report will be a synthesis of the key points from all the FG/ interviews undertaken that week.

GERONTE software developer Company will acknowledge the reports and feedback within 1 working day. Feedback will indicate whether the information contained in the report, is what GERONTE software developer Company require to develop a viable app with the capabilities to fulfil the GERONTE’s project’s goals and meet end-users needs and expectations.

All Partners will share an English translation of their ‘FG/ interview report’ and their ‘summative report’ with all Partners at the end of the FGs.

## Grid 8a. Care pathway design questions

In order to define and detail the care pathway, the overarching objective is to establish the data, health professionals, and communication pathways needed for the Health Professional Consortium to make real patient-centred holistic care and to co-ordinate care accordingly.

The key objective is:

- to develop (explicitly identify key people, support, and communication channels) a pathway that improves the care, and the coordination of care, for older cancer patients with multimorbidity builds on, and integrates with, current systems in a sustainable and adaptable way (future proof)

in a way that keeps what is working well and addresses deficits (as identified based on feedback from patients, carers, clinicians, and literature findings).

*The care pathway design aims and questions* were developed by Diak, University Bordeaux, and University College Dublin based clinicians (referred to as Work Package [WP] 1) following review of the relevant policy, patient and clinician feedback, Grant Agreement, and literature.

| **Preparatory knowledge** | Have stakeholder agreement on the problem and general direction of the solution (e.g. fragmented care and need to integrate care).  Review of the relevant policy, patient and clinician feedback, Grant Agreement, and literature to understand what is feasible aim (or step towards a larger goal). | |
| --- | --- | --- |
| **Aim** | **Question** | |
| **Ideation** | What is your current care path like?  What works well?  What would you like to change?  What would good care look like? | Who provides care in the current system and what care and impact do they have?  Do you have all the information that you need?  What additional information and/ or support do you need?  How do you get information right now?  What would you like to change in relation to the process or outcomes of the information that you get and send?  What makes your current role difficult and what would help you? |
| **User-testing cycle** | Does this care pathway map description provide the functions that you wanted?  Is there anything that you would like to change or add?  What parts of this look user-friendly?  What would you change to make it more user friendly? | |
| **Testing and feedback, or validation cycle** | Does this care pathway map provide the functions that you wanted?  Is there anything that you would like to change or add?  What parts of this look user-friendly?  What would you change to make it more user friendly? | What would you change or add before you put this care pathway into use?  What in your experience would help to make this work well?  What in your experience would be the challenges to making this work well? |

## Grid 8b. Technology design questions

The technology design aims and questions were developed by all GERONTE Partners following review of the relevant policy, patient and clinician feedback, Grant Agreement, and literature.

| **Preparatory knowledge** | Have stakeholder agreement on the problem and general direction of the solution (e.g. fragmented care and need to integrate care).  Review of the relevant policy, patient and clinician feedback, Grant Agreement, and literature to understand what is feasible aim (or step towards a larger goal). | |
| --- | --- | --- |
| **Aim** | **Question** | |
| **Ideation** | What type of information and/ or support do you need more of?  What type of information and/ or support could technology provide for you/ and or your support person?  What features or functions or things would you need to know or have to be comfortable using the technology? | What features/ things would make the technology difficult to use?  Thinking about the technology that you currently have, what is helpful and what is difficult? |
| **User-testing cycle** | Does this technology description or prototype provide the functions that you wanted?  Is there anything that you would like to change or add?  What parts of this look user-friendly?  What would you change to make it more user friendly? | |
| **Testing and feedback, or validation cycle** | Does this technology description or prototype provide the functions that you wanted?  Is there anything that you would like to change or add?  What parts of this look user-friendly?  What would you change to make it more user friendly? | What would you change or add before you put this technology before you put it into use?  What in your experience would help to make this work well?  What in your experience would be the challenges to making this work well? |

| GERONTE Co-design protocol reference list Acocella, I., & Cataldi, S. (2020). Using Focus Groups: Theory, Methodology, Practice. Sage: California.  Agency Clinical Innovation. (2016). Participants experience focus groups: Facilitation Guide. Chatswood: Sydney.  General Data Protection Regulation. (2018). Available at: [General Data Protection Regulation (GDPR) – Official Legal Text (gdpr-info.eu)](https://gdpr-info.eu/). [Accessed 10^th^ September 2021]  Greenhalgh T, Maylor H, Shaw S, Wherton J, Papoutsi C, Betton V, et al. The  NASSS-CAT Tools for Understanding, Guiding, Monitoring, and Researching  Technology Implementation Projects in Health and Social Care: Protocol for  an Evaluation Study in Real-World Settings. JMIR Res Protoc [Internet]. 2020  May 13 [cited 2021 Apr 26];9(5):e16861. Available from: <https://www.researchprotocols.org/2020/5/e16861>  Kruger, R. (2002). Designing and conducting Focus Group Interviews. Minnesota: US  Kruegar, A. & Casey, M. (2014). Focus Groups: A Practical Guide for Applied Research. Sage: California.  Participatory Methods. Institute of development studies. University Susses UK.<https://www.participatorymethods.org/resource/overview-rapid-appraisal-methods-development-settings>  Watts S, Stenner P. Doing Q methodology: theory, method and interpretation.  Qual Res Psychol. 2005;2(1):67–91. |
| --- |

# Roles and tasks in the co-design method

*Note: Table below are labelled to align with the manuscript on this method (published in PLOS ONE journal).*

### Roles and task in the co-design method

Table S5A, S5B, and S5C (below) provide more detail on both the tasks related to the data collection, analysis, and synthesis process, and who is responsible for each task. The full details for these tasks are identified in Grid 7a and 7b.

*How data is collected, analysed, checked, and synthesised*

The researcher:

- collects data identifying end-users/ needs and wants from multidisciplinary FG and or interviews

- rapidly analyses the data from each FG (within 48 hours on the FG taking place for accuracy and support the speed of the design process)

- sends a ‘summary’ of the ‘needs and wants’, as identified in the FG, to the participant for sense-checking (to check the accuracy and completeness).

The participants approve or edit the summary. The researcher synthesises the approved summaries.

*How end-user feedback informs the intervention design*

The synthesised data is sent to the care pathway and/ or design teams.

The design team uses the information to design or refine the care pathway and technology.

The intervention design is presented to the participants at the next cycle.

At the next cycle data is collected, analysed, sense-checked, and synthesised as above.

The design cycles continue until the participants (end-users) approve the design.

###### Table S5A: The researcher’s data collection tasks in the FG

| **FG Preparation** | **Data collection** | **FG records and data analysis process** |
| --- | --- | --- |
| As part of the co-design work, develop the FG/ interview protocol.  The questions will vary in line with the design cycle (such as questions focussed on the ideation, testing, validation) | The researcher organises and runs the FG, including:   - Welcoming and engaging the group - Ensuring consent and setting up relevant recording or note taking mechanism - Introducing the FG aim - **Presenting the questions and facilitating and guiding open** and **inclusive discussion** and follow-up - Closing the session, thank and advising participants on the next steps | Directly after the FG the researcher:  - ensures the security of the FG data, recordings, and all other research related materials  - makes notes on any new points or emphasised points raised in the FG.  Within 24 hours of the **FG the researcher analyses* and develops a** **FG report** on the FG findings.  *The FG *analysis is structured on:*  *- what information did the FG provide or for example and more specifically, ‘what were the participants needs and wants around the care pathway or technology design or user-friendliness*’ |

######

###### Table S5B: The researcher’s tasks in the rapid appraisal and feedback to and from participants

| **Task and responsibility** |
| --- |
| Once the FG summary report is ready, t**he researcher:**   - **Sends the FG report** to the FG participants - Sends instruction on what the researcher is requesting the participant to do   The **FG participant:**   - **receives and reviews FG report** - **confirms** the researcher has ‘interpreted their needs and wants accurately’ or ‘edits the report’ to reflect their needs and wants - Send the edited report back to the researcher   Practical considerations   - Depending on the patient or clinician population the researcher may need to either email the feedback or have a phone call or meet up to discuss the FG report and check it reflect their needs (or edits to do so) - There will be communication between a number of participants, and processes and double-checking are needed to prevent inadvertent sharing of participant contact details across the multiple communications |

###### Table S5C: The researcher’s tasks in the synthesis of data from across FG, sites, and design iterations

| **Task and responsibility** |
| --- |
| The researcher:   - synthesises*** the **confirmed reports** from the ideation cycle of FG - send the ideation cycle synthesised reports to the design team   Notes 1:  - It is practical and reasonable for synthesised FG reports from across a number of sites to be sent to the design team in parallel or sequentially as the design team will develop and refine the design as the data comes in.  ***Data is synthesised in line with the FG questions, design functions and features. The design can be developed and presented in text, model, illustrations in line with what is practical for the care pathway and/ or technology design process.  Note 2:  Cycle 1 is now over and the design team develops a ‘design description, model, prototype’ that the researcher shows to the participants in the second round of FG.  FG data from the 2nd cycle is analysis and synthesis and feedback to the design team again.  The design team develops another more detailed description, model, or prototype, which is then reviewed by the participants in the 3rd design cycle.  The FG data from the 3rd cycle is analysis and synthesis and feedback to the design team again.  If the design met the participants' needs, then no further cycles are necessary.  If further changes are needed, another round of FG and rapid analysis and feedback, and design refinement takes place. |

###### Figure C: Example timeline for the PRA and FG co-design method

The timeline is dependent on a number of factors, which are identified in Table S6 below.

|  | **Month 1** | **Month 2** | **Month 3-4** | | **Month 4-5** | |
| --- | --- | --- | --- | --- | --- | --- |
| **Care**  **pathway** | **1st design cycle**  **Ideation** | | **2nd design cycle**  **User testing and feedback** | | **3rd design cycle**  **Testing + feedback or design validation** | |
|  | **FG** to develop a:  - draft definition of the care pathway.  **Rapid appraisal** to ensure participants' views are reflected in the definition. | The clinician and patient representatives:  - define, detail, and **map the care pathway*** | FG for the end-users to **discuss and give feedback on the:**  **- care pathway map and details** | The clinician and patients:  - develop and **refine the care pathway map** based on the feedback received | FG to:  -review, test, and give further feedback or validate the care pathway map and details | Validated design for the care pathway |
| **Technology** | **1st design cycle**  **Ideation** | | **2nd design cycle**  **User testing and feedback** | | **3rd design cycle**  **Testing + feedback or design validation** | |
|  |  | FG and PRA to **identify the function and feature that the technology needs** to perform to support the care pathway.  The technologist, in collaboration with the clinicians and patient representatives, develop an early prototype of the technology for testing | FG for the end-users to;  **- test and give feedback on the technology’s functions, features, and user-friendliness** | The technologist, in collaboration with the clinicians and patient representatives, **develop and refine the technology** in line with the feedback received | FG to:  -review, test, and give further feedback or validate the technology | Validated design for the technology |

###### Table S6: factors that can impact the time needed across the 3 design cycles

|  | **Number of site and staff needed to work across the sites** | **Consideration** | **Strategies to maximise efficiency in the design timeframe** |
| --- | --- | --- | --- |
| 1 | You are able to conduct FG in the different sites in and around the same time | If you have a large number of sites you may there will be physical and time limits to how many FG you can conduct and how fast you can conduct the rapid analysis and feedback of the FG data | If you have a large number of sites you may then need additional researchers or to extend the time within the design cycles |
| 2 | All sites are able to be included at the same time (recruitment, ethics, staffing, or other factors at individual sites may impact this) | Inclusion of different sites may be staggered across longer times | You may need to extend the time between the design cycles or overlap cycle 1 at site A with cycle 1 at site B (for example). |
| 3 | There are no delays in between sending the feedback to the participants and getting their confirmation or edits back | The tight design cycles is dependant on all part of the FG, rapid analysis, and feedback benign on target (clear and open communication channels supports this) | You may need to extend the time between the design cycles |
| 4 | That each cycle of the design result in sufficient information to develop design for the care pathway and the technology | Effective semi structured FG facilitated by researcher familiar with the project and design process and stage is needed to optimise the direction and output from the FG while being alert to and facilitative of developing relevant data that may emerge | Ensure that you have researcher:  - familiar with the project and design process and stage  - experienced in FG data collection to optimise the direction, interaction, and output from the FG while being alert to and facilitative of developing relevant data that may emerge |
| 5 | There is no delay in the development of the technology | Developing the technology for health care is complex and needs to consider industry standards, integration of the technology and design into the broader system, and relevant data privacy regulations | Ensure that the readiness, resourcing, and health-based experience of the technology developers matches the co-design needs |

#

#

# 
